# Greater medial proximal tibial slope is associated with bone marrow lesions in middle-aged women with early knee osteoarthritis

**DOI:** 10.1186/s10195-023-00739-x

**Published:** 2023-11-28

**Authors:** Hikaru K. Ishibashi, Eiji Sasaki, Kyota Ishibashi, Daisuke Chiba, Takahiro Tsushima, Yuka Kimura, Gentaro Kumagai, Eiichi Tsuda, Kaori Sawada, Tatsuya Mikami, Yasuyuki Ishibashi

**Affiliations:** 1 Department of Orthopaedic Surgery, Hirosaki University Graduate School of Medicine, 5 Zaifu-cho, Hirosaki, Aomori 036-8562 Japan; 2 Department of Rehabilitation Medicine, Hirosaki University Graduate School of Medicine, 5 Zaifu-cho, Hirosaki, Aomori 036-8562 Japan; 3 Department of Innovation Center for Health Promotion, Hirosaki University Graduate School of Medicine, 5 Zaifu-cho, Hirosaki, Aomori 036-8562 Japan

**Keywords:** Early knee osteoarthritis, Bone marrow lesion, Spreading roots sign, Medial proximal tibial angle, Posterior tibial slope, Magnetic resonance imaging

## Abstract

**Background:**

Bone marrow lesion (BML) is an important magnetic resonance finding (MRI) finding that predicts knee osteoarthritis. The purpose of this study was to investigate the influence of proximal tibial morphology on BML, including the spreading root sign (SRS), in women without radiographic knee osteoarthritis (OA). It was hypothesized that varus alignment and a greater posterior tibial slopes (PTS) are associated with BML.

**Materials and methods:**

A total of 359 female volunteers without knee OA who were participants in the Iwaki Health Promotion Project in 2017 or 2019 were enrolled. Participants were divided into the non-OA and early knee OA (EKOA) groups based on the Luyten’s classification criteria. The presence of pathological cartilage lesions, BMLs, attritions, meniscal lesions and effusions was scored on T2-weighted fat-suppressed magnetic resonance imaging (MRI) according to the Whole-Organ MRI Scoring system. The medial proximal tibial angle (MPTA) and medial and lateral PTS (MPTS and LPTS, respectively) were measured. Regression and receiver operating characteristic (ROC) analyses were performed to reveal the relationship between BMLs and proximal tibial morphological parameters.

**Results:**

Of the 359 participants, 54 (15%) were classified as having EKOA. The prevalence of cartilage lesions, BMLs, attritions, meniscal lesions and effusions was higher in the EKOA group than in the non-OA group. The two groups had no significant difference in the proximal tibial parameters. Regression analysis revealed that age and a smaller MPTA were associated with BML in both groups. Attrition (*p* = 0.029) and the MPTS (*p* = 0.025) were positively associated with BML in the EKOA group.

**Conclusion:**

The prevalence of BMLs was higher in women with EKOA and correlated with the varus and greater posterior slopes in those without radiographic knee OA.

***Level of evidence*:**

Level III, retrospective case–control study.

## Introduction

Magnetic resonance imaging (MRI) identifies subtle changes in the cartilage, bone marrow, meniscus and synovium in patients with early knee osteoarthritis (EKOA) before radiological changes appear [1, 2]. Among these changes, bone marrow lesion (BML) is an important finding that predicts knee osteoarthritis (OA) progression [3].

Lower limb alignment has an important role in understanding knee OA aetiology [4, 5]. The Osteoarthritis Initiative cohort reported a significant association between the medial proximal tibial angle (MPTA) and structural progression of knee OA [6]. Additionally, the Iwaki cohort study showed a positive association between BML severity and varus tibial inclination in women with EKOA [7]. However, these reports evaluated only the coronal alignment on plain radiographs. Therefore, the progression of knee OA and the impact of the tibial sagittal plane morphology on the aetiology remain unclear.

The posterior tibial slope (PTS) is a potential risk factor for medial meniscus posterior root tears (MMPRTs) [8–10] and is reportedly associated with the development and progression of knee OA via joint overloading [11–13]. Recently, the spreading root sign (SRS), a type of BML spreading out like roots from the attachment of the medial meniscus posterior root, has been reported to be an important MRI finding in the preliminary stage of MMPRT development [14]. Although this lesion may be affected by intrinsic tibial morphology, its association has not been revealed.

Hence, this study aimed to investigate the proximal tibial morphology and MRI-detected BMLs, including SRS, in Japanese women without radiographic knee OA. It was hypothesized that varus alignment and a greater PTS are associated with BMLs.

## Materials and methods

This study was conducted in accordance with the 1964 Helsinki Declaration and its later amendments and was approved by the ethics committee of Hirosaki University (reference number: 2017-026 and 2019-009). All included participants provided informed consent.

**Fig. 1:**
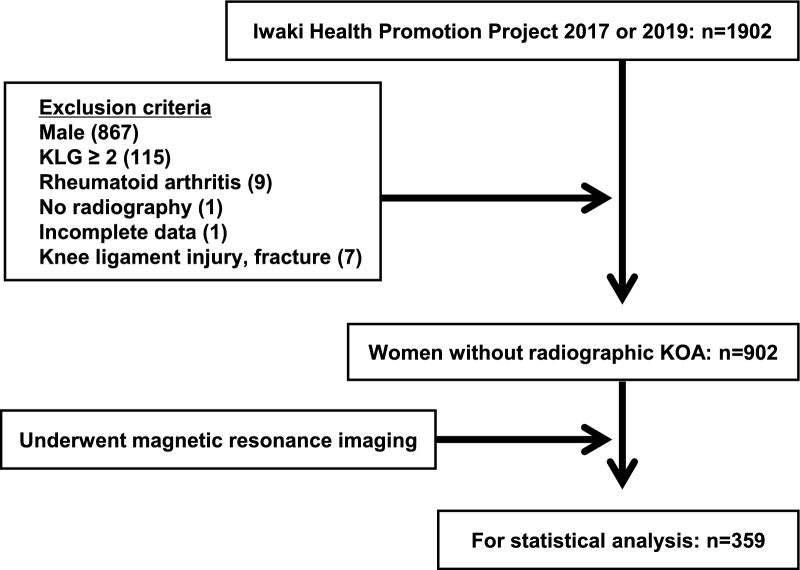
Flowchart illustrating the selection of participants. The participants included in or excluded from the current study are shown. Values in parentheses indicate the number of participants excluded. *KLG* Kellgren–Lawrence grade, *KOA* knee osteoarthritis

All participants of the Iwaki Health Promotion Project, a community-based preventive medicine programme that aims to improve average life expectancy by conducting general health examinations and prophylactic interventions, were included in this study as previously described [15, 16]. In the 2017 and 2019 projects, a total of 1902 volunteers participated. Data of those who participated in both the 2017 and 2019 projects were analysed using the 2017 data. Since the EKOA prevalence is higher in women than in men [17], this study focussed on female participants with no radiographic abnormalities. In total, 1000 participants were excluded for the following reasons: male sex (*n* = 867), radiographic abnormalities [Kellgren–Lawrence (KL) grade ≥ 2, *n* = 115], rheumatoid arthritis (*n* = 9), no radiograph (*n* = 1), incomplete data (*n* = 1), or a history of knee injury (*n* = 7). Female participants without radiographic abnormalities were randomly selected for MRI. A total of 359 participants were included in the final analysis (Fig. 1), and their mean age was 51.3 ± 11.7 years (range 22–85 years) (Table 1).

**Table 1:** Luyten’s classification criteria proposal for early knee OA [18]

A. Patient-based questionnaires (KOOS)
Two out of the four KOOS subscales need to score “positive” (≤ 85%)
1. Pain (9 items, including information on pain intensity, frequency and duration)
2. Symptoms, stiffness (7 items)
3. Function, daily living (short version: 7 items)
4. Knee-related quality of life (QOL; 4 items)

B. Clinical examination
At least 1 criterion needs to be present
Joint line tenderness
Crepitus
C. Radiograph
KL grade 0–I standing, weight-bearing (at least two projections: PA fixed flexion and skyline for patellofemoral OA)

*KOOS* Knee Injury and Osteoarthritis Outcome Score

Questionnaires were administered to investigate lifestyle habits such as habitual drinking, smoking and exercise frequency. Height and weight were measured and body mass index (BMI) was calculated during anthropometric evaluation.

### Radiographic evaluations and bone mineral density

Plain knee radiographs were obtained using the CXDI-40EG digital radiography system (Canon Inc., Tokyo, Japan). Orthopaedic surgeons with over 10 years of experience in radiographic examinations obtained full-extension, weight-bearing and anteroposterior radiographs of both knees with foot map positioning on the day of examination. Sequencing was set at 60 kV, 50 mA and 80 ms for all participants. KL grade ≥ 2 in the most affected knee was defined as knee OA. Two orthopaedic surgeons graded all the joints. Discrepancies were resolved through mutual consultation. Bone mineral density (BMD) at one-third of the distal radius of the nondominant hand was measured on the same day by dual-energy X-ray absorptiometry using the DCS-600EXV bone densitometer (Hitachi Aloka Medical, Tokyo, Japan).

### Classification criteria for EKOA

Participants without knee OA findings on plain radiography were classified into the EKOA group if they met the Luyten’s classification criteria [18] described below; others were classified into the non-EKOA group (Table 1).

- (a) Patient-based questionnaires including the availability of the Knee Injury and Osteoarthritis Outcome Score [19], with at least two of the following required to score positive (i.e. ≤ 85%): pain, symptoms, activities of daily living (short version) and knee-related quality of life.
- (b) Clinical examination required the presence of at least one of the following: joint line tenderness or knee crepitus.
- (c) Radiographs with KL grades of 0 or 1 in the standing and weight-bearing positions.

### Measurements of the proximal tibial morphology using MRI

MRI was performed within 1 week of the other examinations. The right knees were examined using MRI with a rapid extremity coil and a mobile magnetic resonance unit (1.5 T) (ECHELON RX, Hitachi, Tokyo, Japan). MRI was performed in the supine position with fully extended knee. Sequences included sagittal and coronal T2-weighted fat-saturated fast spin-echo (repetition time, 5000 ms; echo time, 80 ms; field of view, 16 cm; 288 × 288 matrices; and slice thickness of 3 mm with a 1.0 mm gap between slices).

**Fig. 2:**
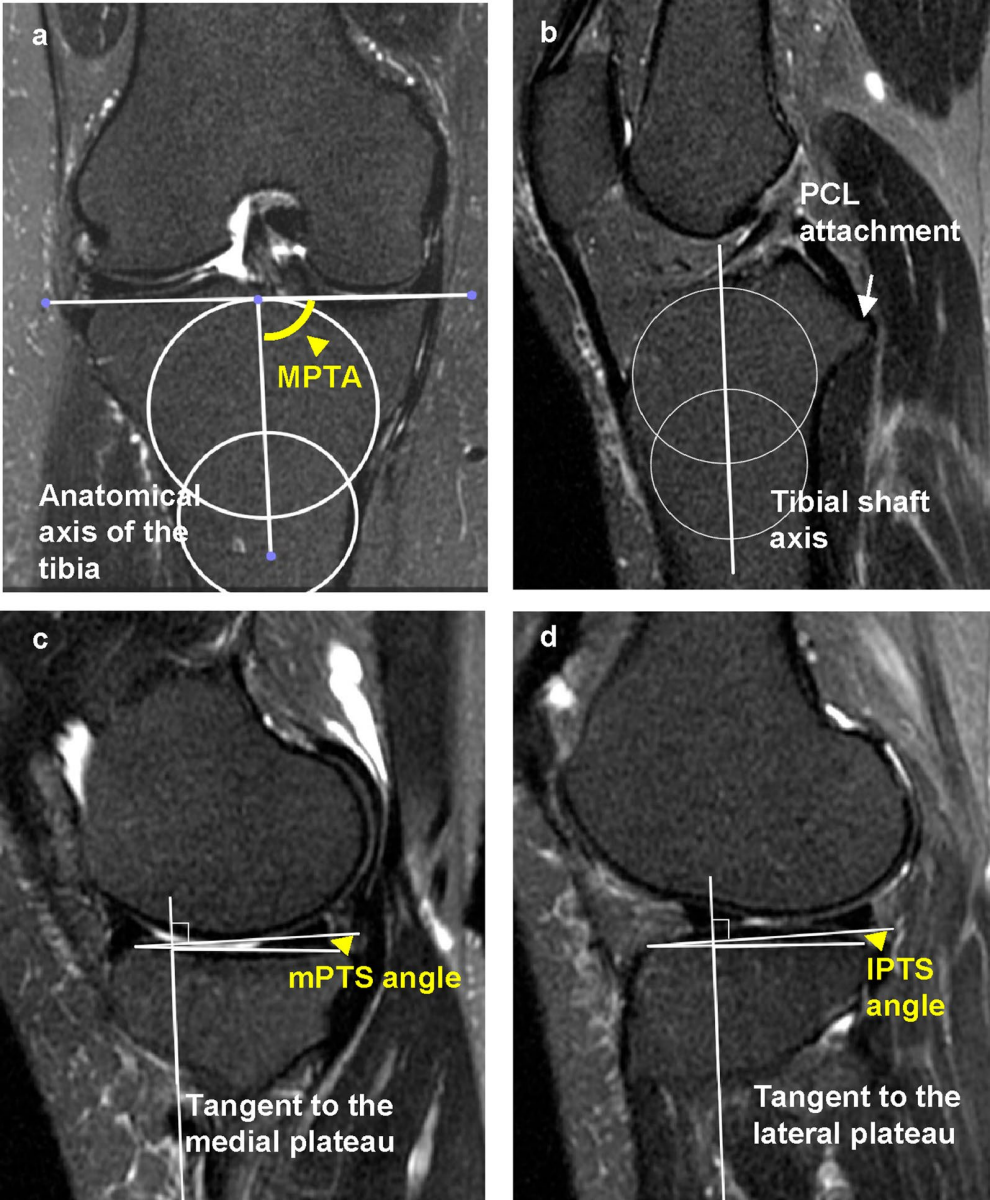
Measurements of the proximal tibial morphological parameters on magnetic resonance images. The MPTA is measured as the angle between the anatomical axis of the tibia and proximal tibial joint line in the coronal view of the MRI image (**a**). The central sagittal plane is determined on the slice based on the attachment of the posterior cruciate ligament and intercondylar eminence. In this slice, two circles fitting the anterior and posterior cortices were placed on the tibia. The line connecting the centres of the two circles was used as the tibial axis (**b**). The MPTS and LPTS are determined by the angle between the axis perpendicular to the tibial axis and the line connecting the two most proximal anterior and posterior subchondral bone points at the centre of the lateral and medial tibiofemoral compartments, respectively (**c**, **d**). *MPTA* medial proximal tibial angle, *MRI* magnetic resonance imaging, *MPTS* medial posterior tibial slope, *LPTS* lateral posterior tibial slope

For the MPTA measurement, a slice was selected that showed the tibial anatomy and medial and lateral menisci in the coronal view of MRI [20]. It was then determined as the angle between the tibial anatomic axis and knee joint line (Fig. 2a). The medial and lateral PTSs were measured as described previously [21]. First, the central sagittal plane was determined on the slice based on the attachment of the posterior cruciate ligament and intercondylar eminence. Within this slice, two circles fitting the anterior and posterior cortices were placed on the tibia. The line connecting the centres of these two circles was used as the tibial axis (Fig. 2b). The medial and lateral PTSs (MPTS and LPTS) were determined as the angle between the axis perpendicular to the tibial axis and the line connecting the two most proximal anterior and posterior subchondral bone points at the centre of the lateral and medial tibiofemoral compartments, respectively (Fig. 2c, d).

Two independent observers examined the interrater reliability of 40 randomly selected images.

### Pathological lesions on MRI

Pathological lesions on the knee joint MRIs were blindly evaluated by two independent observers based on the Whole-Organ MRI Score (WORMS) [22]. Based on the WORMS, synovitis, cartilage damage, BMLs, subchondral cysts, bone attrition, osteophytes and meniscal lesions were evaluated. Synovitis was graded collectively from 0 to 3 on the basis of the estimated maximal distention of the synovial cavity. Regarding cartilage damage, BMLs, subchondral cysts, bone attritions, osteophytes and the number of subregions with scores > 0 were calculated. The meniscal lesions were calculated as the maximum meniscal damage grade of the entire knee. The intra- and inter-rater reliabilities, expressed as interclass correlation coefficients (ICC) (1,1) and (2,1), were 0.929 and 0.921, respectively.

**Fig. 3:**
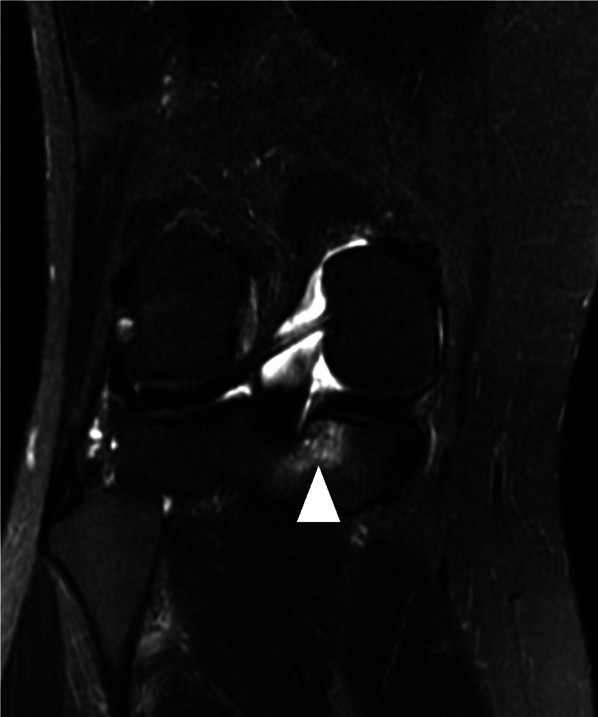
Representative magnetic resonance image showing spreading root sign. Representative SRS images in the coronal plane are shown. High signal intensity is observed in the subchondral bone lesion along with the attachment of the medial meniscus posterior root on T2-weighted fat saturation fast spin-echo images. The white arrow indicates SRS. *SRS* spreading root sign

In addition to the WORMS evaluation, SRS was diagnosed as a BML spreading out like roots from the attachment of the medial meniscus posterior root on the coronal plane of the MRI images [14] (Fig. 3).

### Statistical analysis

Demographic data and proximal tibial morphological parameters in each group are expressed as mean ± standard deviation. The Mann–Whitney *U* and chi-squared tests were performed to compare the two groups because some demographic parameters were not normally distributed by the Shapiro–Wilk test. Proximal tibial morphologies among age groups (< 40, 40–49, 50–59 and ≥ 60 years) were compared using analysis of variance and Tukey’s test. Correlations among the proximal tibial morphologies were evaluated by Spearman’s rank correlation.

Logistic regression analysis was performed with the presence of BMLs as the dependent variable and age, BMI, BMD, lifestyle habits, presence of pathological lesions of cartilage, attrition, meniscal lesions, effusion, MPTA, MPTS and LPTS as independent variables in the non-OA and EKOA groups. These regression models were adjusted for lifestyle habits, including smoking, drinking and fitness. Additionally, to evaluate the associations between proximal tibial morphologies and SRS, logistic regression analysis was performed. SRS was defined as the dependent variable, whereas age, BMI and proximal tibial morphological parameters were defined as the independent variables. To estimate the predictive cut-off level of proximal tibial morphologies for detecting SRS, receiver operating characteristic (ROC) analysis was performed with the MPTA, MPTS and LPTS values as variables. The false-positive fraction was plotted against one true-positive fraction, and the point with the largest slope (closest to the true positive) was the cut-off point. To assess the validity of ROC analysis, the area under the curve (AUC) was calculated. Data inputs and analyses were performed using SPSS version 29.0 (SPSS Inc., Chicago, IL, USA). Statistical significance was set at *p* value < 0.05.

## Results

**Fig. 4:**
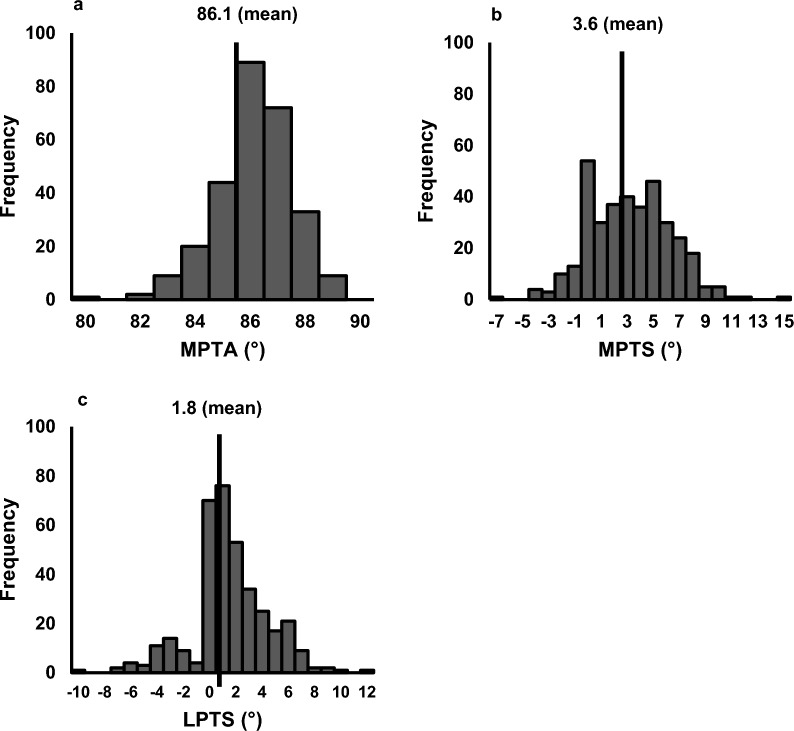
Histograms showing the frequencies of proximal tibial morphologies. Histograms are of MPTA (**a**), MPTS (**b**) and LPTS (**c**). *MPTA* medial proximal tibial angle, *MPTS* medial posterior tibial slope, *LPTS* lateral posterior tibial slope

The mean age of the participants (EKOA group: *n* = 54 and non-OA group: *n* = 305) was 51.3 ± 11.7 years. Demographic data showed no significant differences in BMI, BMD, lifestyle habits and proximal tibial morphological parameters between the two groups (Table 2). The MPTA, MPTS and LPTS were 86.1 ± 1.4°, 3.6 ± 3.3° and 1.8 ± 3.2°, respectively (Fig. 4). The prevalence of cartilage lesions, BMLs, attritions, meniscal lesions and effusions in the EKOA group were 32 (59.3%), 24 (44.4%), 8 (14.8%), 15 (27.8%) and 23 (42.6%), respectively, and was higher than those in the non-OA group (*p* = 0.011, *p* = 0.005, *p* = 0.006, *p* < 0.001 and *p* = 0.002, respectively). In this series, all BMLs were located focally in the femoral condyle or the tibial plateau and none were large. Of the 359 participants, SRS was detected in 5 (1.4%). The MPTA did not significantly correlate with the MPTS (*p* = 0.079) or LPTS (*p* = 0.071). Conversely, the MPTS was positively correlated with the LPTS (*r* = 0.524, *p* < 0.001).

**Table 2:** Clinical characteristics of the participants with and without early knee osteoarthritis

	Total	Non-OA	EKOA	*p*-value
Sample size	359	305	54	
Age, years	51.3 ± 11.7	50.5 ± 11.8	55.9 ± 9.6	0.002
Body mass index, kg/m^2^	21.9 ± 3.1	21.8 ± 3.1	22.5 ± 3.2	0.101
Bone mineral density, g/cm^2^	0.618 ± 0.100	0.619 ± 0.102	0.612 ± 0.093	0.509
Lifestyle habits				
Smoking	33 (9.2%)	28 (9.2%)	5 (9.3%)	0.991
Drinking	136 (38.0%)	115 (37.8%)	21 (38.9%)	0.882
Fitness	87 (24.3%)	71 (23.4%)	16 (29.6%)	0.322
Morphological parameters				
MPTA	86.1° ± 1.4°	86.1° ± 1.5°	86.4° ± 1.3°	0.187
Medial PTS	3.6° ± 3.3°	3.5° ± 3.2°	3.7° ± 3.6°	0.573
Lateral PTS	1.8° ± 3.2°	1.7° ± 3.2°	2.2° ± 3.2°	0.208
MRI findings				
Cartilage lesion	156 (43.5%)	124 (40.7%)	32 (59.3%)	0.011
Bone marrow lesion	103 (28.7%)	79 (25.9%)	24 (44.4%)	0.005
Attrition	23 (6.4%)	15 (4.9%)	8 (14.8%)	0.006
Meniscal lesion	44 (12.3%)	29 (9.5%)	15 (27.8%)	< 0.001
Effusion	93 (25.9%)	70 (23.0%)	23 (42.6%)	0.002
Spreading root sign	5 (1.4%)	4 (1.3%)	1 (1.9%)	0.554

Demographic data and proximal tibial morphological parameters are presented as mean ± standard deviation in the non-OA and EKOA groups. Continuous variables are compared using the Mann–Whitney *U* test, and the ratio of lifestyle habits and the presence of MRI findings are compared using the chi-squared test

*MPTA* medial proximal tibia angle, *PTS* posterior tibia slope, *MRI* magnetic resonance imaging

**Fig. 5:**
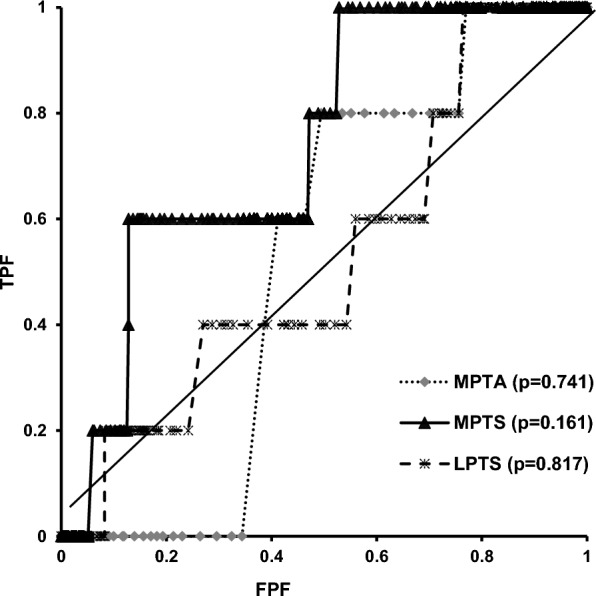
Receiver operating characteristic curve showing the proximal tibial morphological parameters for detecting spreading root sign. ROC curves for MPTA (**a**), MPTS (**b**) and LPTS (**c**). *ROC* receiver operating characteristic, *MPTA* medial proximal tibial angle, *MPTS* medial posterior tibial slope, *LPTS* lateral posterior tibial slope, *TPF* true-positive fraction, *FPF* false-positive fraction, *AUC* area under the curve, *95% CI* 95% confidence interval

Age and the MPTA were positively associated with BMLs (*p* < 0.05) in all the participants by logistic regression analysis. In the non-OA group, the cartilage lesion was positively associated with BMLs (*β* = 0.66, *p* = 0.037). Conversely, attrition (*β* = 8.15, *p* = 0.029) and MPTS (*β* = 0.88, *p* = 0.025) were associated with BMLs in the EKOA group (Table 3). Furthermore, the MPTS was associated with the presence of SRS (*β* = 0.40, *p* = 0.018) (Table 4). ROC analysis revealed that the cut-off values for detecting SRS were 86.1° for the MPTA (AUC: 0.526, *p* = 0.741), 7.2° for the MPTS (AUC: 0.737, *p* = 0.017; sensitivity 0.537, specificity 0.584), and 3.6° for the LPTS (AUC: 0.534, *p* = 0.817) (Fig. 5).

**Table 3:** Factors related to presence of bone marrow lesion in participants with and without early knee osteoarthritis

	Non-OA				EKOA			
	*β*	*p*-value	OR	95% CI	*β*	*p*-value	OR	95% CI
Age	0.05	0.026	1.05	1.01–1.09	0.46	0.018	1.59	1.08–2.324
Body mass index	0.03	0.597	1.03	0.93–1.14	0.04	0.845	1.05	0.67–1.63
Bone mineral density	−3.50	0.122	0.03	0.01–2.54	4.19	0.585	65.75	0.01–2.22 × 10^8^
Cartilage lesion	0.66	0.037	1.93	1.04–3.58	0.74	0.579	2.10	0.15–29.10
Attrition	0.96	0.139	2.61	0.73–9.28	8.15	0.029	3454.54	2.25–5.29 × 10^6^
Meniscus lesion	0.20	0.687	1.22	0.47–3.16	1.35	0.373	3.86	0.20–75.06
Effusion synovitis	0.26	0.442	1.30	0.67–2.54	0.71	0.636	2.03	0.11–38.36
MPTA	−0.36	0.001	0.70	0.56–0.87	−1.72	0.029	0.18	0.04–0.84
Medial PTS	−0.02	0.731	0.98	0.88–1.09	0.88	0.025	2.42	1.12–5.24
Lateral PTS	−0.06	0.301	0.94	0.84–1.06	−0.02	0.935	0.98	0.67–1.44

Logistic regression analysis was performed with the presence of bone marrow lesions as the dependent variable and age; body mass index; bone mineral density; lifestyle habits; presence of pathological lesions of cartilage, namely attrition, meniscal lesions and effusion; medial proximal tibial angle (MPTA); medial posterior tibial slope (PTS) and lateral PTS as dependent variables in the non-OA and EKOA groups. These regression models were adjusted for lifestyle habits, such as smoking, drinking and fitness

*OA* osteoarthritis, *EKOA* early knee osteoarthritis, *CI* confidence interval, *OR* odds ratio

**Table 4:** Relationship between the proximal tibial morphological parameters and presence of spreading root sign

	*β*	*p*-value	Odds ratio	95% CI
Age	0.09	0.167	1.10	0.96–1.25
Body mass index	0.10	0.418	1.11	0.87–1.42
Bone mineral density	10.99	0.118	5.94 × 10^4^	0.06–5.72 × 10^10^
MPTA	−0.13	0.719	0.88	0.42–1.81
Medial PTS	0.40	0.018	1.49	1.07–2.09
Lateral PTS	−0.38	0.071	0.69	0.46–1.03

Logistic regression analysis is performed with the presence of the spreading root sign as the dependent variable, and age, body mass index, bone mineral density, medial proximal tibial angle (MPTA), medial posterior tibial slope (PTS) and lateral PTS as the dependent variables. This regression model is adjusted for lifestyle habits, including smoking, drinking and fitness

*CI* confidence interval

## Discussion

The most important findings of this study were as follows: the EKOA group showed more abnormal MRI findings than did the non-OA group, although there were no significant differences in the proximal tibial morphology. In the EKOA group, greater coronal and sagittal inclinations of the proximal tibia were associated with the presence of BMLs. Furthermore, SRS was found in five (1.4%) healthy female volunteers, and its presence was associated with an increased sagittal inclination of the proximal tibia. The varus deformity in the coronal plane has been shown to be associated with BMLs and progression of OA [6, 7]. This study showed that increased MPTS was also associated with BMLs, which is a risk factor for OA. There have been few reports of such findings in the general community-based volunteer population, and it is our belief that the current study reveals part of the pathogenesis of the progression from EKOA to advanced knee OA. BMLs are pathological lesions that can be detected using MRI and are common findings in patients with both early and advanced knee OA [1, 23]. In histological studies, BMLs represent subchondral microfractures, ischaemia and increased bone turnover [24–26] and are strongly associated with knee symptoms in both the early and end stages of knee OA [27–29]. BMLs predict the progression of structural knee OA, particularly cartilage loss [30]. This study also showed associations between BMLs and cartilage lesions in the non-OA group and between BMLs, attrition and a greater MPTS in the EKOA group. Although a previous study reported an association of varus deformity and BML with elevated serum bone formation markers [7], an association between BMD and proximal tibial morphologies was not detected in the present study.

In the present study, proximal tibial angles did not differ between age groups. This is contrary to the fact that varus alignment progresses in knee OA [31]; however, this may be because the present study excluded participants with advanced knee OA. It could be assumed that patients with varus knee deformities develop knee OA at a younger age, leaving those with a large MPTA in the older age group. Varus knee alignment in the coronal plane [6, 32] and the PTS in the sagittal plane are important factors in the knee OA aetiology [33, 34]. Although mechanical overload on the medial condyles in varus deformities could be a factor in BML development [35], BML may be an irreversible change that improves with a change in knee alignment. Thus, EKOA with BML may be a promising target for preventing knee OA progression before the occurrence of irreversible changes.

SRSs have been considered possible predictors of development of MMPRTs. Nakamura et al. found that the appearance of SRS was consistent with a set of precursory symptoms and recommended SRS as a precursory sign of MMPRTs [14]. Similar to SRS, a posterior shiny-corner lesion (PSCL) was reported to be one of the helpful MRI findings after MMPRTs [36, 37]. Okazaki et al. reported that PSCLs were detected in 64% of patients who underwent surgery for an MMPRT. They concluded that the sensitivity of PSCL was 90.5% within 3 weeks after the occurrence of an MMPRT, and 81.8% within 8 weeks [36]. To the best of our knowledge, this is the first community-based study to report the prevalence of SRS. As the presence of SRS may cause knee symptoms, it is hypothesized that SRS would be detected in knees with EKOA. Unexpectedly, the SRS score was not associated with EKOA in this study, and only five SRS cases were observed, which have been insufficient for statistical analysis. The prevalence may have been low because the study included a general population with non-OA. Future studies may be limited to populations with associated knee pain.

In recent studies, a smaller MPTA and greater PTS have been reported as risk factors for MMPRTs [9, 10]. Okazaki et al. reported the shallow concave shape of the medial tibial plateau as another risk factor for MMPRTs using MRI-based morphometry [8]. A greater PTS and shallow medial tibial plateau increase the anterior tibial translation and shearing force of the medial meniscus [33, 39]. A greater MPTS is also a risk factor for bilateral MMPRTs and may interfere with MMPRTs healing after surgery [9]. These proximal tibial morphologies may cause MMPRTs and result in sequential knee OA. Although the relationship of a greater PTS as a cause of MMPRTs has been well investigated [10, 36], the relationship between SRS and PTS remains unclear. Our study showed that the MPTS was greater in the group with SRS than in the group without SRS. The stress on the attachment of the medial meniscus posterior root caused by a steep PTS may result in SRS and MMPRTs in the future.

The present study had several limitations. First, owing to the exploratory nature of this study, no conclusions regarding the cause-and-effect relationship between BML and PTS and SRS and PTS could be drawn. Participants were regularly followed up to confirm the PTS effect on BML, including SRS. Second, the number of patients with SRS was extremely small in this study. Third, this study was conducted only on women. Owing to the high cost of MRI, it was decided to focus on middle-aged women, the preferred population for examining EKOA. Another limitation was that the BMD was measured at the distal radius instead of the lumber spine and femoral neck, which are the standard diagnosing methods for osteoporosis [27]. Although lower extremity alignment is important for knee OA progression [5, 6], the lack of a full length weight-bearing view was a weak point of this study. Underestimation in procurved tibia may affect PTS. Long-term follow-up will be conducted with the participants of this study to investigate the PTS effect on BML, including SRS and the development of MMPRTs.

## Conclusion

This retrospective study on middle-aged female volunteers without radiographic knee OA found a higher prevalence of BMLs in the EKOA group that correlated with a smaller MPTA and greater MPTS. These results may represent the early pathogenic process of knee OA when no radiographic OA changes have occurred.

## Data Availability

Not applicable.

## Abbreviations

BML: Bone marrow lesions
SRS: Spreading root sign
OA: Osteoarthritis
EKOA: Early knee osteoarthritis
MPTA: Medial proximal tibial angle
MPTS: Medial posterior tibial slope
LPTS: Lateral posterior tibial slope
ROC: Receiver operating characteristic
AUC: Area under the curve
MMPRT: Medial meniscus posterior root tear
PTS: Posterior tibial slope
KL: Kellgren–Lawrence
BMI: Body mass index
BMD: Bone mineral density
WORMS: Whole-Organ MRI score
ICC: Interclass correlation coefficients
PSCL: Posterior shiny-corner lesion
